# Glycerol Production from Glucose and Fructose by 3T3-L1 Cells: A Mechanism of Adipocyte Defense from Excess Substrate

**DOI:** 10.1371/journal.pone.0139502

**Published:** 2015-10-01

**Authors:** María del Mar Romero, David Sabater, José Antonio Fernández-López, Xavier Remesar, Marià Alemany

**Affiliations:** 1 Department of Nutrition and Food Science, Faculty of Biology, University of Barcelona, Av.Diagonal 643, 08028, Barcelona, Spain; 2 Institute of Biomedicine, University of Barcelona, Barcelona, Spain; 3 CIBER Obesity and Nutrition, Barcelona, Spain; East Tennessee State University, UNITED STATES

## Abstract

Cultured adipocytes (3T3-L1) produce large amounts of 3C fragments; largely lactate, depending on medium glucose levels. Increased glycolysis has been observed also *in vivo* in different sites of rat white adipose tissue. We investigated whether fructose can substitute glucose as source of lactate, and, especially whether the glycerol released to the medium was of lipolytic or glycolytic origin. Fructose conversion to lactate and glycerol was lower than that of glucose. The fast exhaustion of medium glucose was unrelated to significant changes in lipid storage. Fructose inhibited to a higher degree than glucose the expression of lipogenic enzymes. When both hexoses were present, the effects of fructose on gene expression prevailed over those of glucose. Adipocytes expressed fructokinase, but not aldolase b. Substantive release of glycerol accompanied lactate when fructose was the substrate. The mass of cell triacylglycerol (and its lack of change) could not justify the comparatively higher amount of glycerol released. Consequently, most of this glycerol should be derived from the glycolytic pathway, since its lipolytic origin could not be (quantitatively) sustained. Proportionally (with respect to lactate plus glycerol), more glycerol was produced from fructose than from glucose, which suggests that part of fructose was catabolized by the alternate (hepatic) fructose pathway. Earlier described adipose glycerophophatase activity may help explain the glycolytic origin of most of the glycerol. However, no gene is known for this enzyme in mammals, which suggests that this function may be carried out by one of the known phosphatases in the tissue. Break up of glycerol-3P to yield glycerol, may be a limiting factor for the synthesis of triacylglycerols through control of glycerol-3P availability. A phosphatase pathway such as that described may have a potential regulatory function, and explain the production of glycerol by adipocytes in the absence of lipolytic stimulation.

## Introduction

Adipose tissue is a critical factor for the control of energy metabolism. Its function is not limited to the adjustment of storage and availability of lipid reserves [1]. Excess food energy intake, largely from fats and small MW carbohydrates, is a main cause of metabolic syndrome [2]. Along this process, adipose tissue plays a critical role [2,3], through its defense against the excess of energy substrates [4].

It is generally assumed that adipocytes, are the most abundant cells (in mass, and probably also in numbers) of most white adipose tissue (WAT) sites [5]. Taking for granted that the adipocyte is essentially a storage bin, albeit with a powerful role in the control and partitioning of energy [6,7], most studies have been devoted to analyze the regulatory side of fat accrual and cytokine control. It is generally assumed that WAT is largely focused on the conversion of glucose into fatty acids (and then triacylglycerols for energy storage), and on the release of fatty acids (and glycerol) from the hydrolysis of acylglycerols [8].

This simple setup established around the synthesis and hydrolysis of storage triacylglycerols is often related with the purported disrupting effects of hypoxia on WAT, constituting one of the key factors in the development of inflammation [9], a cause/ consequence of the derangement of WAT metabolic and control functions [10,11]. A large body of evidence sustains these postulates; however, careful studies have shown that, *in vivo*, WAT consumes very little oxygen [12]. Mature cultured 3T3-L1 cells have proven to be practically anaerobic, producing large amounts of lactate at the expense of medium glucose [13] under normoxic conditions. Lactate production *in vivo* has been observed in different WAT sites under basal conditions and under a cafeteria diet [14]. In any case, the large lactate production depends on glucose availability [15,16] and may be part of a mechanism to dispose of excess glucose and cytoplasmic NADH.

In order to check whether the over-active WAT glycolytic activity is specific of glucose, we devised a simple experiment in which 3T3-L1 adipocytes, free of fibroblasts, were incubated in media with increasing levels of glucose and/or fructose. We also analyzed the release of glycerol, another 3C metabolite, in parallel to lactate, as a way to dispose of glucose carbon and maintain cytoplasmic NAD^+^/NADH balance, and as a part of a mechanism to control the synthesis of acylglycerols.

## Materials and Methods

### Cells and differentiation

3T3-L1 cells (ATCC CL 173) were obtained from ATCC (Manassas, VA USA), and stored under liquid nitrogen; they were used at a passage not higher than 6. The cells were cultured in 6-well plates (Costar 3506, Corning Life Sciences, Corning, NY USA) with 24 mm polyester membrane Transwell inserts (Corning Life Sciences), under standard conditions, as previously described [17]. We used DMEM GlutaMAX-I (Gibco Life Technologies, Roche Diagnostics, Indianapolis, IN USA), supplemented with 10% newborn calf serum (NBS; Gibco Life Technologies), 25 mM Hepes, 100 U/mL penicillin and 100 mg/L streptomycin (all from Sigma-Aldrich, St Louis MO USA). Two days post confluence, the cells were differentiated to adipocytes using the same medium containing 10% fetal bovine serum (FBS, Gibco) instead of NBS, supplemented with 5 mg/L insulin, 250 nM dexamethasone and 0,5 mM IBMX (all from Sigma-Aldrich). Two days later, the new medium was supplemented only with insulin; and after two more days, the cells were maintained in the standard medium (DMEM 21063–029, containing 25 mM glucose and no phenol red, Gibco) with no added hormones, and was changed every 2 days. Under the conditions used, pH was 7.4–7.8. Cells were incubated at 37°C in an oven ventilated with air supplemented with 5% CO_2_ that gave a theoretical pO_2_ of 20 kPa (i.e. 0.2 mM dissolved O_2_). The pCO_2_ was in the range of 5 kPa, corresponding to 1.7 mM dissolved CO_2_ [17].

### Experimental design

The study was carried out during three days. The basal medium was identical to the standard one, but devoid of glucose (DMEM 11966–025, no glucose, Gibco). It was supplemented with 5% FBS and, especially glucose (glc) and/or fructose (frc). [0 mM glc + 0 mM frc], [2.75 mM glc + 2.75 mM frc], [5.5 mM glc + 0 mM frc], [0 mM glc + 5.5 mM frc], [11 mM glc+ 0 mM frc], [0 mM glc + 11 mM frc], [5.5 mM glc + 5.5 mM frc], [22 mM glc + 0 mM frc] and [0 mM glc + 22 mM frc]. The media (final volume 1.5 mL) were replaced daily. Spent media were frozen and stored for the analysis of metabolites. After the last medium drainage, the cells were harvested.

### Cell harvesting

The cells were harvested using trypsin (Sigma-Aldrich) and mechanical separation, following the protocol for trypsinization in Transwell inserts established by the provider (Corning Life Sciences). Under these conditions, the cultures contained only plurivacuolar adipocytes, with nil presence of fibroblasts (checked by microscope observation).

Harvested cells were suspended in 1 mL NBS (adjusted with the established sugar(s) concentration), and then their number and size distribution were measured with a portable cell counter, Scepter Handheld Automated Cell Counter (PHCC20060 Scepter, Merck Millipore, Billerica, MA USA) as previously described [17]. The Scepter used the Coulter technology, measuring particle sized through electrical resistance changes as it crosses a pore. The total volume of particles counted was considered "total biomass", whilst that of particles within the 18–24 μm diameter range were considered 3T3-L1 adipocytes [18] and their total number and volume ("cell volume") as well as mean cell volume were calculated from the Scepter count data. The differences between the sum of cell volumes and “biomass” (i.e. cell debris) was in the 9–12%, range of total biomass, with no significant differences between groups.

Cells' total RNA was extracted using the GenEluteTM (RTN, from Sigma-Aldrich) isolation procedure, and DNAase (Sigma-Aldrich), following the instructions of the provider. RNA was later used for gene expression analysis.

Adipocyte triacylglycerol content ranges between 70 and 85% [19]; since 3T3-L1 cells were plurivacuolar, we assumed that their lipid content was 75% at most. Based on this approximate Fig, and a cell fat density of about 0.89 g/mL we were able to estimate that 1 mmol of triacylglycerol (standard rat lipid composition) corresponds to 997 μL. The application of these values to the sum of cell volumes in each well gave us an approximate estimation of the amount of lipid these cells contained, and which is also known to be essentially made up of triacylglycerols.

### Analyses of the media

The spent media were used for the analysis of glucose (kit 11504, Biosystems, Barcelona Spain); lactate (kit 1001330 Spinreact, Sant Esteve d'en Bas, Spain); glycerol (F6428 free glycerol reagent, Sigma-Aldrich); and free fatty acids (NEFA-HR 434–91795, Wako, Neuss, Germany). Fructose+glucose (i.e. total hexose) was measured with a kit (ab83380, Abcam, Cambridge UK), the concentrations of fructose were calculated from the corresponding hexoses and glucose data. For each well, all analyses were carried out in duplicate using standards prepared using the basal (zero glucose) incubation medium (including both glucose and fructose).

### Gene expression analyses

After counting, cells were suspended in a lysis buffer. Total RNA from harvested cells in different experiments was quantified using a ND-100 spectrophotometer (Nanodrop Technologies, Wilmington DE USA). RNA samples were reverse transcribed using the MMLV reverse transcriptase (Promega, Madison, WI USA) and oligo-dT primers. Real-time PCR amplification was done using 10 μL amplification mixtures containing Power SYBR Green PCR Master Mix (Applied Biosystems, Foster City, CA USA), equivalent to 4 ng of reverse-transcribed RNA and 150 nM primers. Reactions were run on an ABI PRISM 7900 HT detection system (Applied Biosystems) using a fluorescent threshold manually set to 0.150 for all runs. Table 1 shows the primers used for the estimation of gene expression; the *Rpl32* gene was used as control of charge.

**Table 1:** Genes (and their corresponding proteins, and primer sequences used) which expression was analyzed in mature 3T3-L1 cells incubated in the presence of varying concentrations of glucose and fructose.

gene	protein	EC num	5' > 3'	3' > 5'	bp	M
*Glut1*	glucose transporter 1	-	GCTTCCTGCTCATCAATCGT	CTTCTTCTCCCGCATCATCT	134	+
*Glut4*	glucose transporter 4	-	TTCTATTTGCCGTCCTCCTG	ACTGGGTTTCACCTCCTGCT	140	+
*Glut5*	glucose transporter 5	-	GGCTCATCTTCCCCTTCATT	ATGTCCTGCCCTTGGTCTC	123	-
*Aqp7*	aquaporin 7	-	TTTTGCCACCTATCTTCCTGA	GCTGGACTGTTCTTCTTGTCG	120	+
*Hk1*	hexokinase 1	2.7.1.1	ACGGGACGCTCTACAAACTC	ACAGGAGGAAGGACACGGTA	96	+
*Hk2*	hexokinase 2	2.7.1.1	CTCTCTCAACCCTGGCAAAC	GCACAATCTCGCCCAAGTA	68	+
*Khk*	fructokinase (ketohexokinase)	2.7.1.4	GAGGTGGTGTTTGTCAGCAA	ATGAGCGTAGCCCCTTTCTT	107	+
*Pfkl*	6-phosphofructokinase (liver type)	2.7.1.11	CCAATGCTCCAGACTCAGC	AGATTCAGCCACCACTGCTC	130	+
*Pfkm*	6-phosphofructokinase (muscle type)	2.7.1.11	TGGTGCTGAGGAATGAGAAA	TCAAAGGGAGTTGGGCTTC	145	+
*Aldoa*	aldolase a (muscle type)	4.1.2.13	TCAAGTCCAAGGGTGGTGTT	TGGGTAGTTGTCTCGCCATT	85	+
*Aldob*	aldolase b (liver type)	4.1.2.13	TCCAAGAAAATGCCAATGCT	GGCTCAACAATAGGGACCAG	76	-
*Dak*	triokinase (dihydroxyacetone kinase 2 homolog)	2.7.1.28	CAAGACTGACCTCCCAACCT	CTGCCCACAGAGAATCCAG	113	+
*Adh4*	alcohol dehydrogenase type 4	1.1.1.1	GCCATCAATACTGCCAAGGT	AGGCCAAAGACAGCACAAGT	53	+
*Adh7*	alcohol dehydrogenase type 7	1.1.1.1	GGACTCTACCAAGCCCATCA	GATGAGAGGGCATCAACCAT	111	+
*Gpd1*	glycerol-P dehydrogenase type 1	1.1.1.8	GGAGATGCTAAATGGGCAGA	GCAGTGAACAAGGGGAACTT	105	+
*Gyk*	glycerol kinase	2.7.1.30	GGGTTGGTGTGTGGAGTCTT	AACGGATTTCGCTTTCTTCA	96	+
*Acp1*	acid phosphatase (soluble)	3.1.3.2	GCTGTTCGTGTGTCTCGGTA	CCGCACTGTCTATCCTCCA	110	+
*Acp2*	acid phosphatase (lysosomal)	3.1.3.2	GAGCCTGTCATACCCAAGGA	GGATGGTGAGGAGCAACACT	133	+
*Acp5*	acid phosphatase (tartrate-resistant)	3.1.3.2	CATACGGGGTCACTGCCTAC	CACTCAGCACATAGCCCACA	87	+
*Alpl*	alkaline phosphatase (liver type)	3.1.3.1	GGTGAACGGGAAAATGTCTC	GTGAAGCAGGTGTGCCATC	141	+
*Aldh1a1*	aldehyde dehydrogenase type 1	1.2.1.3	GTCAAGCCAGCAGAGCAAA	AATGTTTACCACGCCAGGAG	90	+
*Aldh2*	aldehyde dehydrogenase type 4	1.2.1.3	GTGTTCGGGGACGTAAAAGA	TTGAGGATTTGCATCACTGG	74	+
*Gpam*	glycerol-P acyl-transferase	2.3.1.15	TCCAGTTCCCGAGTCTGAGT	TTTTCACAGCGTTCTTCACG	122	+
*Lpl*	lipoprotein lipase	3.1.1.34	GCCAAGAGAAGCAGCAAGAT	CCATCCTCAGTCCCAGAAAA	101	+
*Hsl*	hormone-sensitive lipase	3.1.1.79	CTGCTTCTCCCTCTCGTCTG	CAAAATGGTCCTCTGCCTCT	108	+
*Atgl*	triacylglycerol lipase (adipose-type)	3.1.1.3	CAAACAGGGCTACAGAGATGG	AAGGGTTGGGTTGGTTCAGT	68	+
*Ldha*	lactic acid dehydrogenase (muscle type)	1.1.1.27	GCATCCCATTTCCACCAT	TCCGAGATTCCATTTTGTCC	96	+
*Ldhb*	lactic acid dehydrogenase (heart type)	1.1.1.27	GATTCACCCCGTGTCTACCA	AGCGACCTCATCGTCCTTC	136	+
*Pdk4*	pyruvate dehydrogenase kinase type 4	2.7.11.2	ACCGCATTTCTACTCGGATG	CTTGGGTTTCCCGTCTTTG	73	+
*Fas*	fatty acid synthase	2.3.1.85	AGAGGCTTGTGCTGACTTCC	AATGTGCTTGGCTTGGTAGC	59	+
*Acc1*	acetyl-CoA carboxylase	6.4.1.2	GGAGCCAGAAGGGACAGTAGA	CAGCCAAGCGGATGTAAACT	92	+
*Acly*	ATP: citrate lyase	4.1.3.8	GATGAAGAAGGAGGGGAAGC	GGGAAGTGCTGTTTGACGA	111	+
*Cpt1b*	carnitine palmitoyl-transferase b	2.3.1.21	CGCAGGAGGAAGGGTAGAGT	CCAGGGTCACAAAGAAAGCA	110	+
*Rpl32*	ribosomal protein L32 [housekeeping gene]	-	CTGGAGGTGCTGCTGATGT	GGGATTGGTGACTCTGATGG	123	+

The column M indicates whether the gene was adequately transcribed, a—sign indicates that the number of cycles at which the expression was observed was too high and the response was not linear.

A semiquantitative approach for the estimation of the concentration of specific gene mRNAs per cell or unit of tissue weight was used [20]. The data were presented as the number of transcript copies per cell, allowing for direct comparisons independently of the number of cells in a given well.

### Statistics

Statistical comparisons using 2-way anova were done with the Prism 5 (GraphPad Software, San Diego CA USA) graphics/ statistics package. Three-way anova analyses were done with the Statgraphics Centurion XVI program package (Statpoint Technologies, Warrengton, VA USA).

## Results

### Cell number and size

Table 2 shows the final cell counts, measured mean cell volume and total “biomass” (i.e. cells plus debris) in each incubation well, as well as an estimation of the triacylglycerol content of the cells in each well. There were no significant differences in cell size with respect to the parameters of initial monosaccharide concentration in the medium and its type. The proportion of debris with respect to cells was not significantly different either.

**Table 2:** 3T3-L1 cell volumes and estimated triacylglycerol content after 3-day exposure to media with different glucose or fructose concentrations.

glucose	fructose	cells/well	cell volume	well biomass	debris	cell TAG	ΔTAG
mM	mM	x10^3^	pL	μL	%	pmol/well	pmol/day
0	0	664 ± 24	4.64 ± 1.31	3.51 ± 1.16	12	3.5	0.00
5.5	0	838 ± 48	5.29 ± 1.10	4.89 ± 1.36	9	4.0	+0.49
11	0	848 ± 6	5.11 ± 1.78	4.75 ± 1.19	9	3.8	+0.35
22	0	1018 ± 46	4.66 ± 1.16	5.38 ± 1.28	12	3.5	+0.02
2.75	2.75	843 ± 30	5.10 ± 1.63	4.84 ± 1.13	11	3.8	+0.35
5.5	5.5	891 ± 2	5.32 ± 1.95	5.20 ± 1.75	9	4.0	+0.51
0	5.5	818 ± 66	5.09 ± 2.22	4.68 ± 1.54	11	3.8	+0.34
0	11	832 ± 26	4.77 ± 1.92	4.45 ± 1.28	11	3.6	+0.10
0	22	776 ± 18	4.46 ± 0.52	3.96 ± 1.07	13	3.4	-0.13

The data represent the mean ± SD of the analyses of three different (contiguous) wells with Transwell inserts. Well biomass represents the sum of volumes of all particles detected; the differences between cell volumes and total biomass is presented as well debris (as a percentage of total biomass). Cell triacylglycerols (TAG) were estimated, as described under Methods, assuming that at most 75% of the cell volume was made up of TAG. The estimations are an approximation made using only the mean values, as are the changes observed in biomass per cell (comparing the volumes of cells with those in the medium devoid of monosaccharides. The differences in cell volume (mass) were translated into estimated changes of triacylglycerol content per day. There were no statistically significant differences for cell volume, or type and concentration of monosaccharide (3-way ANOVA)

**Fig 1:**
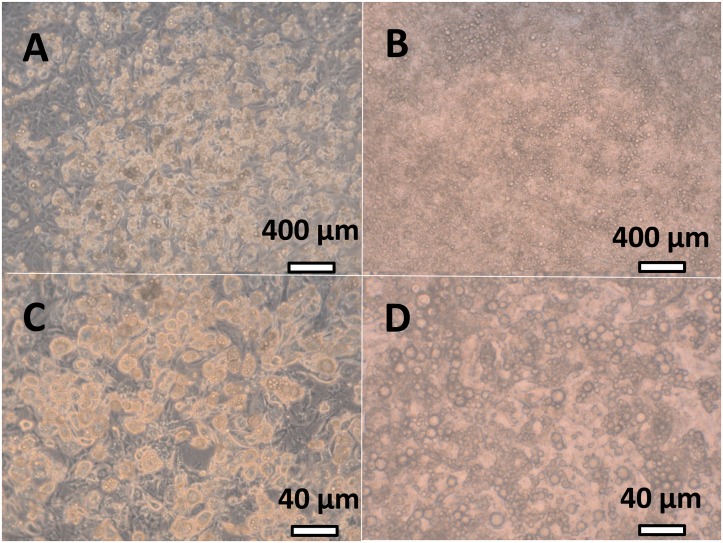
Microphotographs of 3T3-L1 cells in culture. The cells shown were maintained under the conditions described in the text. They are representative of the conditions used throughout the whole study. Photographs A and B are shown at a lower magnifying power than C and D. A white bar shows a reference for size. Panes A and C correspond to an ordinary 3T3-L1 culture, in which differentiated adipocytes and undifferentiated fibroblasts coexist. Panels B and D represent 3T3-L1 cultures incubates using Transwells, as described for this study. All wells used in this investigation were maintained under these same conditions; there was a total absence of undifferentiated fibroblasts, as previously described [17]

Fig 1 depicts an example of standard 3T3-L1 cells two days after the beginning of the incubation in the presence of 11 mM glucose, compared with a standard 3T3-L1 cell culture incubated with the same medium but not using Transwells [17]. There was a marked absence of fibroblasts under the conditions of incubation used, in comparison with their common presence in the standard culture,

### Glucose and fructose utilization

**Fig 2:**
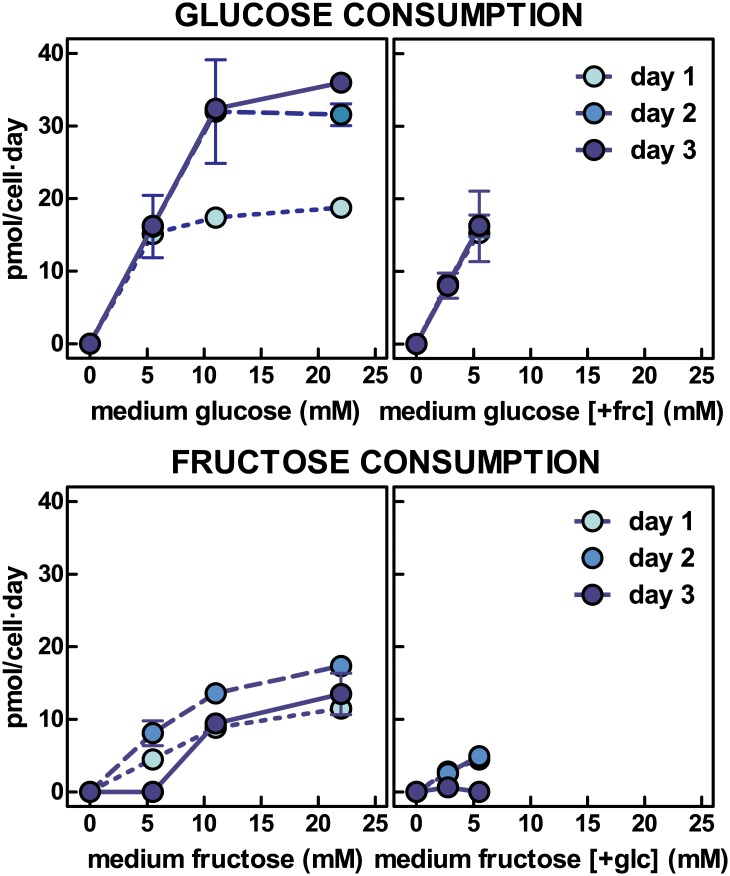
Glucose and fructose consumption by 3T3-L1 adipocytes exposed to a medium with varying concentrations of glucose, fructose or a combination of both. Each dot represents the mean ± sem of three different wells. Glucose or hexose consumption are expressed in pmol per cell and day. There were statistically significant (two-way anova) differences (P<0.05) for 11 mM and 22 mM glucose absorption data on day 1 vs. days 2 and 3 (the latter only for 22 mM glucose). There were significant differences for fructose vs. glucose consumption for concentrations higher than 5.5 mM and for days 1 to 3. Fructose consumption in the presence of glucose was also statistically different from that of glucose in the presence of fructose for 2.75 mM to 5.5 mM initial medium levels. In the graphs where glucose + fructose were present in the medium, the X-axis legend shows the hexose measured, and the other present but not shown in the graph is represented between brackets.

Since the final counts of cells per well were variable, the data on glucose or fructose consumption (i.e. decrease in medium sugar levels) were corrected by the number of cells. Fig 2 shows the rates of consumption of glucose and fructose per cell and day. Glucose consumption increased in proportion to the initial glucose added up to 11 mM, stabilizing thereafter. In fact, these data correspond to a consumption higher than 77% of glucose in the medium, up to 11 mM. This Fig corresponds to the maximal capacity of the cells to use glucose as substrate through glycolysis. The presence of added fructose did not change the efficiency of glucose handling. However, there was a significant difference in the rates of glucose utilization with time: the limit of consumption was lower (in the range of 20 pmol/cell·day) on the first day; but on the second and third, the rates practically doubled. The rates of consumption of medium fructose were much lower, and tended to decrease with a prolonged exposure to fructose. The data presented suggest that the combination of glucose and fructose resulted in the practical exhaustion of glucose, with only a minimal (albeit variable) amount of fructose used.

### Lactate and glycerol release into the medium

**Fig 3:**
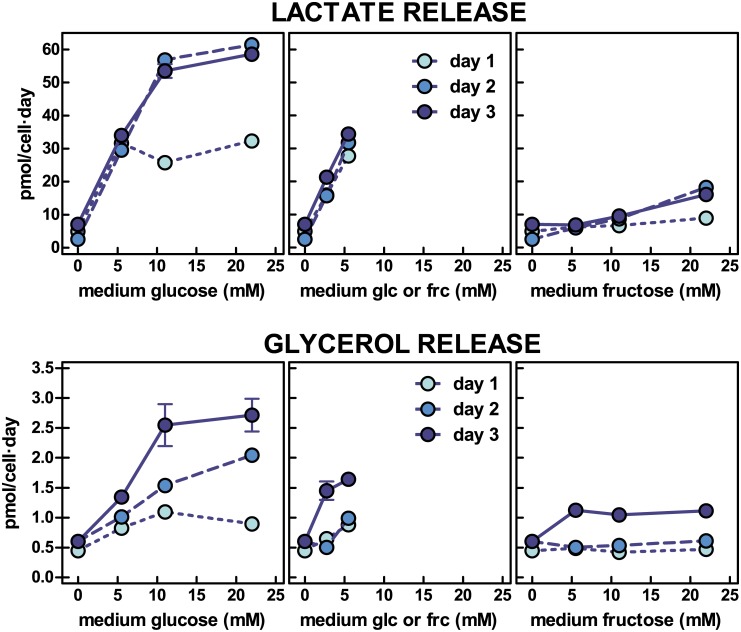
Lactate and glycerol efflux from 3T3-L1 adipocytes exposed to a medium with varying concentration of glucose, fructose or a combination of both. Each dot represent the mean ± sem of three different wells. Lactate or glycerol release are expressed in pmol per cell and day. There were statistically significant (two-way anova) differences (P<0.05) for lactate release at 11 mM and 22 mM glucose on day 1 vs. days 2 and 3. There were significant differences for concentrations from 5.5 mM onwards in the production of lactate from medium fructose compared with glucose for all three days. Glycerol release showed significant differences for 22 mM glucose on days 1 vs 2 and 3 and for 11 mM glucose for day 1 vs day 3. There were significant differences between glycerol release from fructose vs. glucose at 11 mM and 22 mM for days 2 and 3. In the graphs where glucose + fructose were present in the medium, the X-axis legend shows the concentration of each of both hexoses.

The fate of the glucose taken from the medium can be easily deduced from Fig 3, where the appearance (production rates) of lactate in the medium was parallel to the rates of glucose consumption, showing, again, lower rates for day 1. The production of lactate from fructose was lower, and stabilized to a level, which was also lower than that obtained with glucose.

At a concentration of glucose 11 mM, on days 2 and 3, in an incubation well, the cells consumed practically all glucose available, with most of the hexose carbon being released as lactate. In the case of fructose, under comparable circumstances, the consumption of the hexose was lower than 40% of that available, and the production of lactate justified even less than this proportion. Fig 3 also shows the rates of glycerol release to the medium. When the initial substrate was glucose, the proportionality of glycerol release with respect to the initial concentration of glucose was maintained; however, the rate of glycerol release with time doubled on the third day of the study with respect to the data measured in the first. This effect of the length of exposure was also observed when the substrate was fructose, but in this case, the maximal rate observed was about 1.3 pmol/cell·day, compared with 2.7 pmol/cell·day for glucose.

The mean ratios of carbon lactate production *vs*. carbon glucose consumed were 0.97, and 0.40 for fructose when using only the 11 mM and 22 mM values for calculation (P<0.05). The corresponding data for glycerol were 0.038 (glucose) and 0.028 (fructose), the differences being not statistically significant. However the 3C metabolite secreted concentrations ratio: (glycerol/lactate) was 0.035 for glucose and 0.080 for fructose (P<0.05) when the hexoses were analyzed separately and not including those mixed in the medium.

Analysis of the presence of non-esterified fatty acids in the medium (which contained about 0.5 g/L albumin) gave, in all cases, values below the limit of detection of the method, with no differences related to hexose concentrations (data not shown)

### Gene expression data: Carbohydrate and pyruvate metabolism

**Fig 4:**
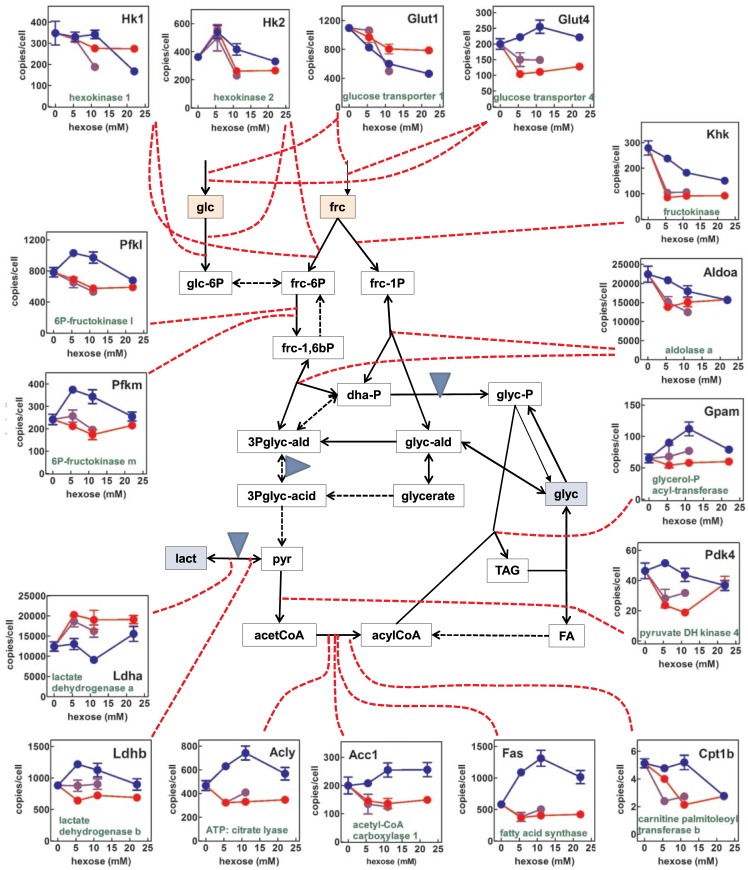
Gene expression of enzymes of glucose/ fructose metabolism and triacylglycerol synthesis in 3T3-L1 adipocytes exposed to a medium with varying concentrations of glucose, fructose or a combination of both. Solid lines represent the paths analyzed in this Fig and in Fig 4; blue triangles indicate the sites of production and utilization of NADH. The graphs indicate the number of copies of specific mRNAs for the genes (in black) corresponding to the enzymes and transporters (in green) acting on the corresponding paths (linked by red dotted lines). In each graph, the expressions in the presence of glucose are marked in blue, and those with fructose in red. The mixed-hexose groups are marked in purple. Each point corresponds to the mean ± sem of three different wells. Abbreviations: glc = glucose; frc = fructose; dha-P = dihydroxyacetone-P; glyc = glycerol; glyc-ald = glyceraldehyde; glyc-acid = glycerate; lact = lactate; pyr = pyruvate; TAG = triacylglycerols; acetCoA = acetyl-CoA; FA = fatty acids. The statistical significance of the differences between groups presented here correspond to Figs 3 and 4. The effect of hexose was analyzed with two-way anova: glucose or fructose *vs*. concentration (in this case, for the sake of clarity, the mixed hexose groups were not included in the analysis). The differences between glc and frc groups were significant (p<0.0001) for all genes except *Hk2* (P = 0.0190), and *Aldoa* (0.0011) and not significant only for *Adh4*. The effect of "concentration" was analyzed using one-way anova analyses for glucose, fructose and their mixture. For glucose, all genes responses were significant (P values between 0.05 and 0.0001) except *Glut4*, *Ldha*, *Acc1*, *Pdk4*, *Aldh2*, *Lpl*, *Acp5* and *Aqp7* (NS). Changes of gene expression with fructose concentration were all statistically significant except for *Hk1*, *Pfkm*, *Acc1*, and *Gpam* (NS). In the case of glc+frc, all genes responded significantly to hexose concentration except *Glut4*, *Pfkm*, *Ldhb*, *Acc1*, *Fas*, *Gpam*, *Pdk4*, *Aldh1a1*, *Atgl*, *Hsl*, and *Acp2*

Fig 4 presents a composite scheme of the changes in the expression of selected genes of glucose oxidation and lipid metabolism in 3T3-L1 cells subjected to different concentrations of glucose and fructose in the medium. *Glut5* (glucose transporter 5) expression was not detected, and thus not included in the graph. The expression of *Glut1* was higher than that of *Glut4*, and their patterns were different. *Glut1* expression was higher with fructose than with glucose, and in both cases, the expression decreased with growing medium monosaccharide. *Glut4* reversed this pattern; showing higher (and maintained with increasing glucose levels) expressions for glucose. Fructose decreased its expression markedly with little change in function of hexose concentration. Both hexokinases gene (*Hk1* and *Hk2*) expressions tended to decrease with increasing hexose concentrations, with significant differences between fructose and glucose for *Hk1*, but not for *Hk2*. We found a robust basal expression of the fructokinase gene (*Khk*), inhibited by fructose; its expression also decreased with increasing concentrations of glucose.

Expressions of phosphofructokinase genes (*Pfkl* and *Pfkm*) showed a similar pattern; with maximal expression in the presence of glucose as compared with fructose; expression peaked at the 5.5–11 mM range. No detectable expression of the aldolase b (liver type) gene (*Aldob*) was found (data not shown in the Fig). However, muscle type aldolase gene (*Aldoa*) was highly expressed, showing the general pattern of decreasing its expression with increasing initial levels of glucose or fructose, with no differences between them.

The 3T3-L1 cells showed a more marked expression of muscle-type lactate dehydrogenase (*Ldha*) than of the heart type (*Ldhb*) genes, but they showed reversed patterns. *Ldha* increased its expression with the presence of fructose, and was practically unchanged by glucose (nadir at 11 mM), whilst *Ldhb* increased with glucose and was unchanged by fructose. Pyruvate oxidation by pyruvate dehydrogenase was estimated indirectly from the expression of the gene for pyruvate dehydrogenase kinase 4 (*Pdk4*), which controls the activity of the pyruvate dehydrogenase complex. Increases in glucose levels practically did not change the expression of *Pdk4*, which was deeply depressed by fructose in a biphasic way (lowest peak at 5.5–11 mM), suggesting that, within a physiological range of concentration, glucose maintained pyruvate dehydrogenase inhibited, but fructose did not.

### Gene expression data: Lipid metabolism

The genes controlling the main enzymes participating in the synthesis of fatty acids: ATP: citrate lyase (*Acly*), acetyl-CoA carboxylase 1 (*Acc1*) and fatty acid synthase (*Fas*) showed comparable patterns. Increases in glucose levels raised their expressions, and fructose did not change them (or decreased them slightly) in the absence of hexose. When both glucose and fructose were present, the direction of change in most gene expressions was marked by the presence of fructose: the fructose effects prevailed over those of glucose

The transfer of acyl-CoA to mitochondria was probably very limited, since the expression of carnitine-palmitoleoyl transferase 1 (*Cpt1b*) was the lowest of those analyzed; glucose maintained unchanged its level of expression except for a decrease observed at 22 mM, whilst fructose decreased steadily the gene expression, with a nadir at 11 mM. For that gene, the “fructose effect" was again present.

The glycerol kinase gene (*Gyk*), which showed an already low expression, was further decreased by increasing levels of glucose or fructose, the effects of the latter being more marked. The expression of the glycerol transporter aquaporin 7 (*Aqp7*) was maintained unchanged with increasing glucose levels, up to 22 mM, when decreased, however, fructose induced a marked decrease in the expression of the gene (down by 2/3rds), a trend already observed at 5.5 mM.

The expression of the gene for glycerol-3P acyl-transferase (*Gpam*) was unaffected by fructose, either alone or combined with glucose; but glucose, at 11 mM, increased its expression to a maximal value, decreasing again at 22 mM.

The expression of the genes for the three main adipose tissue lipases: lipoprotein lipase (*Lpl*), adipose type triacylglycerol lipase (*Atgl*) and hormone-sensitive lipase (*Hsl*) was more robust than that of genes controlling lipogenesis (Fig 4). The pattern was similar, with slight differences for all three genes: increase in expression along with increasing glucose levels in the medium, and lack of change or slight decrease with increasing fructose, resulting in an overall inhibiting effect of fructose.

### Gene expression data: Glycerol metabolism

**Fig 5:**
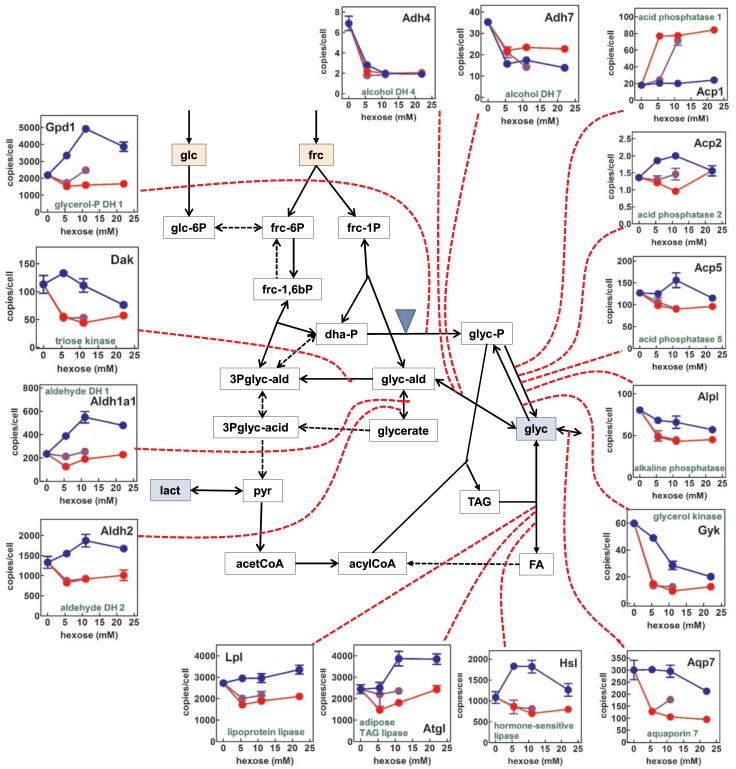
Gene expression of enzymes of glycerol metabolism in 3T3-L1 adipocytes exposed to a medium with varying concentrations of glucose, fructose or a combination of both. Graphical conventions and abbreviations are the same as in Fig 3. The graphs indicate the number of copies of specific mRNAs for the genes (in black) corresponding to the enzymes and transporters (in green) acting on the corresponding paths (linked by red dotted lines). In each graph, the expressions in the presence of glucose are marked in blue, and those with fructose in red. The mixed-hexose groups are marked in purple. The short names used in this graph are the same as in Fig 3. The statistical significance of the differences between groups is presented in the legend to Fig 3.

Fig 5 presents a scheme of the pathways implicated and the expressions of the main enzymes related to the synthesis and utilization of glycerol in mammals. This Fig continues and complements Fig 4, which included the pathway for incorporation of glycerol to acylglycerols, its main fate in adipose tissue.

Alcohol dehydrogenases gene (*Adh4* and *Adh7*) expressions were low and tended to decrease with increasing monosaccharide concentrations to a plateau from 5.5 mM onwards. This contrasts with the expression of aldehyde dehydrogenases (*Aldh1a1* and *Aldh2*), with higher number of copies per cell and a similar pattern: increased gene expression with increasing glucose levels, rising to a plateau from 11 mM onwards, and maintained or slightly decreased expression when fructose was present in the medium, irrespective of its concentration. This pattern was also closely followed by *Gpd1*, glycerol-P dehydrogenase (type 1), which again showed an inhibiting effect of fructose. The expression of triose kinase (*Dak*) was maintained under low glucose levels, decreasing progressively from a peak at 5.5 mM, but fructose (with or without the presence of glucose) reduced its expression by half from 5.5 mM on.

We also measured the expression of four phosphatases, described in murine tissues and able to hydrolyze glycerol-3P, since (as far as we know) no mammalian gene has been associated with the glycerol-phosphatase activity (otherwise well known for decades). We selected three acid and one alkaline phosphatases. The *Acp1* gene was unaltered by glucose but its expression increased four-fold with fructose. *Acp2* was expressed with a lower number of copies and, similarly to *Acp5* (the phosphatase with higher level of expression), showed practically unchanged expressions under fructose and slightly increased with high levels of glucose, as usual, peaking at 11 mM. Alkaline phosphatase gene (*Alpl*) tended to decrease its expression in the presence of hexoses, the effect being more marked with fructose.

## Discussion

Adipocytes can use fructose as metabolic substrate [21] with independence of insulin-controlled glycolysis, since in addition to hexokinase they express fructokinase. However, the efficiency of hexose uptake and/or catabolism along the glycolytic pathway was less effective for fructose than for glucose. Fibroblasts can grow and differentiate in media with fructose, which can later be used for lipid synthesis [22], and the production of lactate, as observed here.

Since the expressions of both glucose transporters and hexokinases did not show marked differences on the effects elicited by glucose or fructose, it seems improbable that the limiting step observed here in the utilization of fructose by the adipocyte would be the control of their uptake and incorporation. In fact, the presence of fructokinase may favor (in theory) the incorporation of fructose. However, the absence of *Aldob* and the preference of aldolase a (*Aldoa* gene) for fructose 1,6 bis-P rather than the product of fructokinase: fructose-1-P, make improbable the massive processing of fructose through the parallel (typical of liver) pathway channeling half the fructose C to glyceraldehyde. Thus, we can assume that the specific fructose pathway of liver [23] is present, but not fully operative in WAT.

The main problem of the theoretically easy entry of fructose into the cell is how to explain the wide differences in lactate production (and on gene expression) observed when glucose or fructose (or both together) were used as substrates. Once fructose is phosphorylated to fructose-6P, it is undistinguishable of that formed from glucose-6P via phophoglucomutase. Therefore, the differences can be explained only by additional effects of fructose at the regulatory level, marking the difference with glucose. The effective blockage by fructose on the expression of lipid metabolism genes compounds these effects. This is in agreement with the inhibition by excess dietary fructose on the control of liver substrate metabolism [24]; this question remains to be explored in depth. On the other hand, the lack of intensive fructose catabolism (as compared to glucose by 3T3-L1 cells) seems to help rule out a direct effect of fructose increasing the accumulation of fat in WAT, in spite of its marked obesogenic effects alone [25] or in combination with excess dietary lipid [26].

WAT breaks up glucose to lactate in order to decrease glucose levels and export 3C units to other tissues as energy substrate, or to the liver for lipogenesis, oxidation or gluconeogenesis, completing a Cori cycle [27]. Lactate fate will depend on hepatic energy availability and the presence of glucose, which would inhibit the gluconeogenic processing of lactate [28]. The fact that 3T3-L1 adipocytes produced less lactate when exposed to fructose suggests that the ultimate reason for WAT lactate production is not the simple utilization of the glycolysis-derived ATP (as in the Warburg effect) [29]. Instead, the extensive break-up of glucose may be just a mechanism to help maintain glycaemia and/or to prevent the toxicity of excess glucose [30], including the adipocyte itself.

The main strength of this study is the combination of quantitative data of substrate conversion into 3C metabolites and an extensive analysis of gene expressions representative of interconnected pathways using concordant (i.e. same well) data. The limitations are those inherent to the use of a cell culture, i.e. the extent of concordant genomic or proteomic analyses because of the size of the sample, as well as the absence of dynamic studies (such as tracers or gene silencing). The focus on genomic data allowed a perhaps more quantitative approach, but at the expense of better knowledge of protein levels, identification and activity.

**Fig 6:**
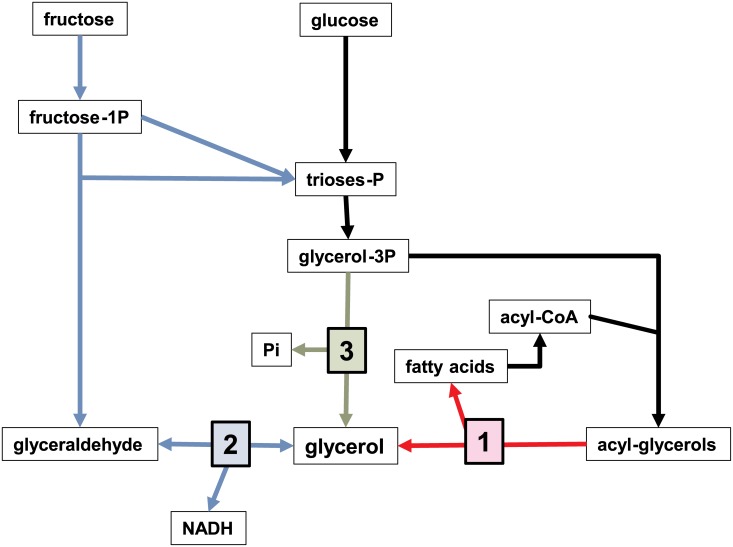
Glycerol-producing pathways in mammalian adipocytes. The numbers indicate the pathways described in the Discussion: 1: lipolysis (red); 2: reduction of fructose-derived glyceraldehyde (exclusive fructose metabolism: blue); 3: postulated glycerol-3-phosphatase pathway (green).

Probably, the most critical finding of this study is the quantitatively important release of glycerol by WAT in response to increasing medium hexoses. The net and quantitatively significant release of glycerol to the bloodstream [31], irrespective of its lipolytic or glycolytic origin, provides the liver with an excellent (and non-acidic) gluconeogenic substrate, which is directly incorporated at the triose-P level. Fig 6 shows the three known pathways for the production (and release) of glycerol into the medium by mammalian adipocytes. Path 1 is the best-known, glycerol as a byproduct of lipolysis [32]; there are, however, studies indicating a significant contribution of the glycolytic pathway to the production of glycerol [33].

The amount of glycerol present in the wells, consequence of the metabolic action of the cells was about two orders of magnitude higher than the glycerol contained in the cells' triacylglycerol lipid droplets. When cells were exposed to 11 mM glucose, the net release rate of glycerol per well was (third day) 2.5 μmol/day. The amount of triacylglycerol (within the cells and debris) per well was in the range of 4 pmol. The comparison of these two Figs clearly indicates that the complete turnover (i.e. hydrolysis and re-synthesis of all triacylglycerols) in one day could, at most, produce 4 pmol glycerol, almost six orders of magnitude lower than the actual glycerol measured in the medium. In spite of the inaccuracy of the method used to estimate the lipid content of 3T3-L1 cells in each well, the differences in magnitude are overwhelming, in no way, so much glycerol could have been produced by lipolysis. Moreover, even if it were the case, the lack of changes in biomass reinforce this conclusion. Lastly, where were the fatty acids released by that massive lipolysis? It is highly improbable that a massive oxidation occurred, first because of the limited entry of substrates into the mitochondria, and second because of the impossibility of coexistence of lactate production, to export reducing power, and an active oxidative metabolism. However, overall, the absence of free fatty acids in the medium proved the biochemical impossibility of lipolysis as provider a large portion of so much glycerol in the medium.

It could be expected that the cells maintained at about 0 mM hexoses would use stored triacylglycerols for energy, thus releasing more glycerol as a byproduct. This was not the case either. The experimental data clearly indicate that the production of glycerol was a timed response to the challenge of excess glucose, and to a lesser extent, fructose, essentially unrelated to the stored triacylglycerol mass and its eventual turnover or hydrolysis.

Pathway 2 [34] has been relatively unexplored; the lack of specificity of alcohol and aldehyde dehydrogenases do not rule out this possibility, but the reaction most favored is in the direction of glycerol oxidation, rather than its synthesis because of a high K_M_ [35].

In any case, glycerol formation has an advantage similar to that of lactate: the disposal of excess cytoplasmic NADH produced by triose-P dehydrogenase, with the added benefit of lack of acidity and the inconvenience of net loss of ATP production compared with lactate. This path assumes that glycerol is formed from glyceraldehyde by reduction with NADH [36]. For this pathway to be effective it needs a sustained source of glyceraldehyde, such as that released by aldolase b acting on fructose-1P (the product of fructokinase) [37], which makes it almost exclusive of fructose catabolism via its alternative pathway.

However, two factors suggest that in WAT, this pathway may have a (probably minor) contribution to the production of glycerol. First is the presence of fructokinase, which produces fructose-1-P, split by aldolases (mainly aldolase b) into dihydroxyacetone-P and glyceraldehyde. The latter needs either to be oxidized to glycerate and phosphorylated by triokinase to 3P-glyceric acid to enter the glycolytic pathway [38], or to be reduced to glycerol by alcohol dehydrogenases [34]. Second, the ratio of lactate *vs*. glycerol produced from fructose alone was almost twice that obtained from glucose. This difference cannot be justified only by the lower use of fructose as substrate, pointing to the probable contribution (albeit minor) of fructose-derived glyceraldehyde to the production of glycerol. Nevertheless, these data agree with a limited capacity of oxidation of fructose by adipocytes [39,40].

Neither path 1 nor path 2 can explain the large release of glycerol from 3T3-L1 adipocytes incubated in the presence of high hexose concentrations. Thus, we explored Path 3. The activity of glycerol-3P phosphatase has been described in mammals, but no gene has been attributed to that enzyme activity. A plausible explanation is the limited specificity of many phosphatases, which may act on cytoplasmic glycerol-3P. Earlier enzymologists have even used the term "glycerophosphatase" to describe both acid and alkaline phosphatases, because both enzymes can hydrolyze glycerol-3P [41,42]. The lack of a gene in mammals marks a clear difference with yeasts, plants and bacteria, where glycerol synthesis is an important mechanism of survival [43]. The reason for this difference may be that the postulated phosphorolysis of glycerol-3P is carried out by one or more phosphatases already described, or the hydrolysis is carried out by a phosphatase so far unknown. We analyzed the expression of four probable phosphatase candidates (because they are relatively unspecific, their abundance and general distribution) found in murine tissues, as described in Figs 4 and 5. The similar pattern of *Acp2* and *Acp5* expression (*vs*. medium glucose) with those of the main lipogenic control genes *Acc1* and *Fas*, was in line with that parallelism, but we lack further proof.

The close relationship of glycerol-P phosphatase and the synthesis of lipid was postulated as early as 1939 [44], but this line of study was not continued. The increase in glycerol-3-P induced by the activation of the glycolytic pathway favors the synthesis of acylglycerols if paired with increased synthesis of acyl-CoA. Thus, high glycerol-P production is expected to proceed in parallel to lipogenesis when there is a high availability of glucose. The limitation of glycerol-3P availability may be part of the adipocyte defense mechanism against exposure to excess substrate availability. The possible regulation, by a phosphatase of the levels of glycerol-3P would represent an obvious advantage for the regulation of acylglycerol synthesis, dependent on glycerol-3P [45]. A phosphatase-catalyzed reduction of (high glucose-induced) glycerol-3P levels may decrease the synthesis and storage of triacylglycerols. These relationships are already paralleled in gluconeogenesis [46,47]. We are aware that this assumption is, right now, largely speculative, and needs a slow and extensive enzymological analysis to prove. The ubiquitous presence of phosphatases [48], and the finding of "glycerol-phosphatase" activity in adipose tissue [49] seem to suggest, however, that probably one (or more) of the selected phosphatases does indeed participate in the regulation of glycerol-3P levels, and, consequently help control the synthesis of acylglycerols. A number of changes have been observed in alkaline phosphatase with obesity and diet [50,51], but so far, these activities have not been related with the core of the synthesis of triacylglycerols. Unfortunately, the data available could not pinpoint which could be the main phosphatase implicated in the process. However, because of location, known functions and capability of regulation, we assume that this regulatory role may correspond to a non-lysosomal acid phosphatase, probably *Acp2* or *Acp5*, widely distributed in WAT, since their stimulation by medium glucose concentration is not related to their purported functions in the cell.

It is possible that the postulated (albeit not proven) phosphatase pathway will eventually provide more insight on the control of lipogenesis in adipocytes, and becoming a potential target for the treatments of excess accumulation of fat.
